# Breaking the lead barrier: subcutaneous implantable cardioverter-defibrillator implantation in Ebstein anomaly with left ventricular noncompaction

**DOI:** 10.1016/j.hrcr.2026.03.023

**Published:** 2026-04-03

**Authors:** Mohamed Shelig, Alhasan Alzubi, Davinder Singh, Alec Fletcher, Ashraf Elghul, Khalid Abozguia

**Affiliations:** 1 Department of Internal Medicine, Marshall University Joan C. Edwards School of Medicine, Huntington, West Virginia; 2 Department of Cardiology, Marshall University Joan C. Edwards School of Medicine, Huntington, West Virginia

**Keywords:** Ebstein anomaly (EA), Left ventricular noncompaction (LVNC), Subcutaneous implantable cardioverter-defibrillator (S-ICD), Congenital heart disease, Ventricular tachycardia (VT), Sudden cardiac death

**Key Teaching Points**

• The co-occurrence of Ebstein anomaly and left ventricular noncompaction (LVNC) cardiomyopathy creates significant anatomical barriers to conventional transvenous implantable cardioverter-defibrillator (ICD) placement, including risk of tricuspid valve damage, lead instability in a small functional right ventricle, and potential perforation of the friable noncompacted myocardium.
• Subcutaneous ICD is a viable and effective alternative for secondary prevention of sudden cardiac death in patients with complex congenital heart disease where transvenous lead placement poses unacceptable risk, particularly in younger patients who may require multiple device revisions over their lifetime.
• A negative electrophysiology study does not diminish the indication for ICD implantation in patients with documented sustained ventricular tachycardia (VT) and LVNC, as inducibility of monomorphic VT during electrophysiology studies in this population is reported to be as low as 8%.
• When echocardiographic windows are suboptimal, cardiac magnetic resonance imaging serves as the definitive imaging modality for diagnosing LVNC, using the Petersen criterion (noncompaction-to-compaction ratio > 2.3 at end-diastole) to confirm the diagnosis and guide further management.

## Introduction

Ebstein anomaly (EA), a congenital malformation of the heart, is characterized by downward apical displacement of the tricuspid valve with adherence of the posterior and septal leaflets to the myocardium. In addition, there is atrialization and dilatation of the inlet portion of the right ventricle. Presentation of this defect varies, but often results in the development of tricuspid regurgitation, right ventricular failure, and arrhythmias.^1^^,^^2^ The prevalence of tachyarrhythmias is common because of the increased incidence of accessory atrioventricular pathways, which are reported to exist in nearly a quarter of patients with EA.^3^ Rarely, ventricular arrhythmias may also occur in these patients, often necessitating placement of an implantable cardioverter-defibrillator (ICD).

Left ventricular noncompaction (LVNC) is a rare cardiomyopathy in which the myocardial architecture is “spongy” with prominent trabeculations and deep intertrabecular recesses. This cardiomyopathy occurs because of arrested myocardial compaction during embryonic development.^4^ Although various arrhythmias have been noted to occur in these patients, ventricular tachyarrhythmias are the most prevalent, contributing to sudden cardiac death.^5^

The co-occurrence of EA and LVNC presents unique challenges regarding conventional transvenous ICD implantation. The abnormal structure of the tricuspid valve and right ventricular anatomy, in addition to the friable noncompacted myocardium, creates substantial risks of lead malposition, perforation, and device-related complications.

We present the case of a 41-year-old patient with EA and LVNC who successfully received subcutaneous ICD after a conventional transvenous ICD was deemed unsuitable because of structural complexities, highlighting the clinical decision-making process and therapeutic outcomes in this challenging population.

## Case presentation

**Figure 1:**
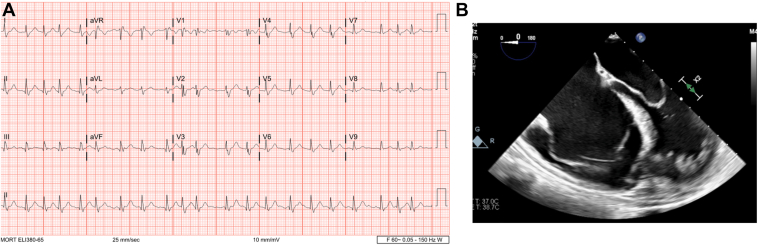
**A:** Electrocardiogram demonstrating atrial fibrillation with rapid ventricular response. **B:** Apical 4-chamber echocardiographic view showing features of Ebstein anomaly. Note the massively enlarged right atrium, atrialized right ventricle with a small functional chamber, and apically displaced tricuspid valve leaflets (hallmark finding).

A 42-year-old man with a history of hypertension and diabetes mellitus was evaluated by emergency medical services after an episode of central chest pain, presyncope, and palpitations associated with sweating and clamminess. At the scene, the patient was found to be in sustained monomorphic ventricular tachycardia (VT) with a rate exceeding 200 beats/min. The patient underwent cardioversion once, with the subsequent rhythm showing atrial fibrillation with rapid ventricular response. Upon arrival to the emergency department, the patient’s electrocardiogram revealed atrial fibrillation with rapid ventricular response with a heart rate of 127 beats/min, without significant ST-segment deviation (Figure 1A). Consecutive high-sensitivity troponin levels were 88, 359, and 443 ng/L respectively.

**Figure 2:**
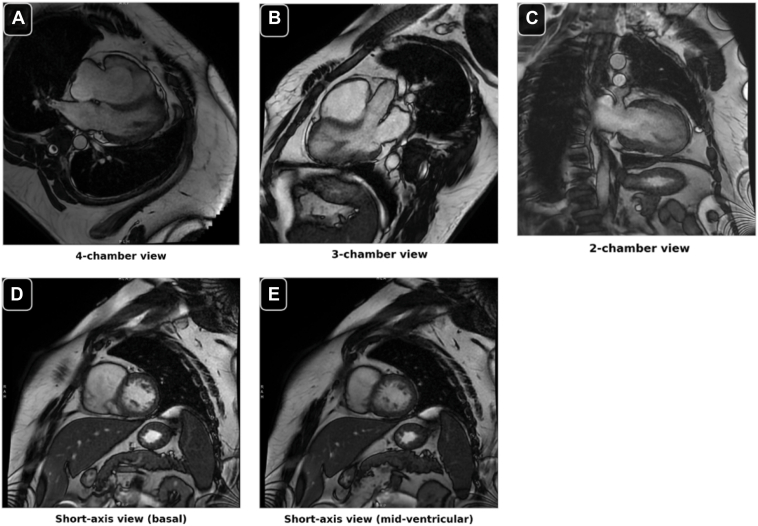
Cardiac magnetic resonance imaging demonstrating left ventricular noncompaction (LVNC). (**A**) 4-chamber view, (**B**) 3-chamber view, (**C**) 2-chamber view, and (**D and E**) short-axis views at basal and mid-ventricular levels. Prominent trabeculations are seen involving the mid-ventricle to apex, with a noncompacted-to-compacted myocardial ratio exceeding 2.3/1 at end-diastole, fulfilling the Petersen cardiac magnetic resonance diagnostic criterion for LVNC.

The patient was initially managed with heparin and diltiazem infusion, subsequently reducing his heart rate to the 80-beat/min range. The absence of accessory pathway conduction during atrial fibrillation was confirmed by electrocardiographic morphology, which showed absence of preexcitation, allowing safe use of diltiazem. This was subsequently verified by the negative electrophysiology (EP) study, which demonstrated no evidence of an accessory pathway. The echocardiogram revealed normal global systolic left ventricular function with an ejection fraction of 52%. In addition, prominent trabeculations were noted, concerning for possible LVNC cardiomyopathy. The tricuspid valve was poorly visualized and noted to be displaced toward the apex, consistent with EA, with mild tricuspid regurgitation. The right ventricular cavity was noted to be small (Figure 1B). Unfortunately, the study was technically difficult because of the patient’s body habitus. The next morning, our patient underwent left heart catheterization, revealing normal coronary arteries, ejection fraction, and left ventricular end-diastolic pressure. Reversible causes were ruled out with normal urine drug screen and thyroid function test results. Given these findings, further evaluation was prompted. Cardiac magnetic resonance imaging was completed, which revealed excessive left ventricular trabeculations of the mid-ventricle to apex, which can be seen with noncompaction cardiomyopathy (Figure 2). The images revealed a noncompaction/compaction ratio exceeding the Petersen diagnostic criterion of >2.3/1 at end-diastole.^6^ Subsequently, an EP study was conducted, which was negative, with no evidence of an accessory pathway, no inducible supraventricular or VTs, and no dual atrioventricular nodal physiology.

**Figure 3:**
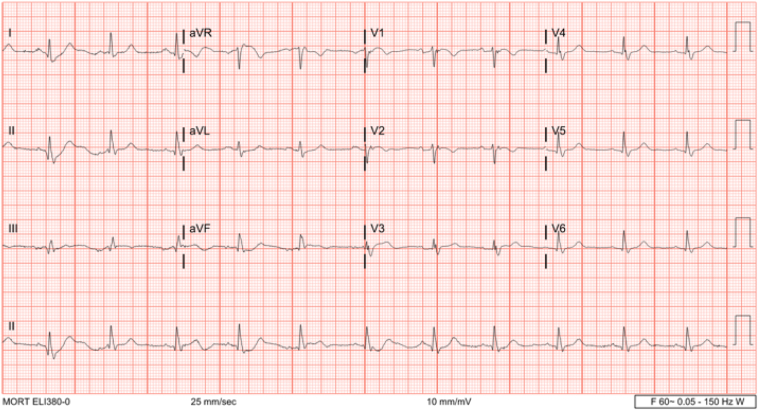
Baseline electrocardiogram demonstrating sinus rhythm.

Given the patient’s presentation with wide-complex tachyarrhythmia, near syncope, and noncompaction cardiomyopathy, the decision was made to implant an ICD. After discussion of the case with an adult congenital heart disease specialist and with the patient, a shared decision was made to proceed with a subcutaneous ICD implant in lieu of a conventional ICD. The baseline electrocardiogram demonstrated sinus rhythm with no evidence of bundle branch block (Figure 3). Implantation of a subcutaneous ICD was successful, and 10-J defibrillation testing was performed with satisfactory impedance (75 Ω). The patient was discharged with metoprolol succinate 12.5 mg daily.

At follow-up, device interrogation demonstrated normal function, with no events noted. The patient has been doing well, with no recurrence of initial symptoms. He was able to resume driving and return to work.

## Discussion

This case illustrates the successful application of subcutaneous ICD technology in the context of congenital heart disease co-occurring with LVNC cardiomyopathy. Specifically, this highlights the clinical decision-making process involved when traditional transvenous ICD placement is considered high risk. The co-occurrence of EA and LVNC cardiomyopathy created a particularly challenging scenario that required careful consideration of both anatomical constraints and arrhythmic risk stratification.

Our patient’s presentation with monomorphic VT at a rate exceeding 200 beats/min, accompanied by hemodynamic compromise evidenced by presyncope, established the need for ICD placement for secondary prevention.^7^ While EA is more commonly associated with supraventricular tachycardias, the concurrent diagnosis of LVNC significantly elevated the patient’s risk for developing recurrent ventricular arrhythmias. In patients with LVNC alone, the risk of ventricular tachyarrhythmias is reported as occurring in nearly half of those affected, with sudden cardiac death being the major cause of mortality.^5^

The EP study conducted on the patient was negative for reproducible arrhythmia and accessory pathways, which may initially seem contradictory to the clinical presentation. However, it is worth noting that this finding is not uncommon in patients with LVNC. In a study conducted by Steffel et al,^8^ in which EP studies were conducted in patients with isolated LVNC, sustained monomorphic VT was inducible in only 8% of patients. Hence, the negative EP study in our patient did not diminish the indication for ICD implantation for secondary prevention, given the documented event of sustained monomorphic VT on presentation.

When considering ICD implantation in this patient, we encountered a complex decision regarding device selection. EA complicates traditional transvenous ICD placement because the tricuspid valve is apically displaced and part of the right ventricle is “atrialized,” meaning the functional right ventricle is small and distorted. A transvenous lead risks having its coil cross the tricuspid valve and sit largely in the atrium—potentially damaging the valve, increasing tricuspid regurgitation, reducing defibrillation efficacy, and subjecting the lead to mechanical stress and instability. Published reports confirm that in this population, transvenous leads are associated with worse morbidity, higher likelihood of valve dysfunction, and more frequent reinterventions.^9^ Further studies have demonstrated increased morbidity with lead placement in this population, with an increased need for reintervention.^10^^,^^11^

Similarly, among those with LVNC cardiomyopathy, there are challenges associated with lead placement. The presence of friable noncompacted myocardium potentially poses a risk of perforation and unstable lead positioning. In addition, the spongy architecture of the myocardium may result in suboptimal defibrillation thresholds and unreliable sensing. There is a gap in the literature regarding complications of ICD lead placement in patients with LVNC, and further studies are needed.^12^

While this case demonstrates successful subcutaneous ICD placement in a patient with underlying congenital and structural cardiac abnormalities, long-term follow-up is essential to evaluate device performance, including appropriate shock delivery and any late complications. As the use of subcutaneous ICD devices in congenital heart disease and cardiomyopathy continues to grow, this technology may become increasingly important in managing high-risk arrhythmias in anatomically challenging hearts, where a conventional ICD lead may pose an elevated complication risk.

## Conclusion

This case demonstrates the successful use of subcutaneous ICD technology in a patient with congenital heart disease and cardiomyopathy, where implantation of a traditional transvenous ICD posed significant anatomical and technical challenges. The rare co-occurrence of EA and LVNC cardiomyopathy created a unique clinical scenario requiring careful multidisciplinary clinical decision making and risk-benefit analysis to ensure the best possible outcome for the patient. Implantation of a subcutaneous ICD provided an effective solution for secondary prevention of sudden cardiac death while avoiding potential risks associated with transvenous ICD placement. Future studies with larger patient cohorts and long-term follow-up data are essential in solidifying the role of subcutaneous ICD technology for secondary prevention within this patient population.
